# Effectiveness of Granulocyte Transfusions in Neutropenic Adult Oncology Patients: A Comprehensive Review of the Literature

**Published:** 2016-05-01

**Authors:** Asha Demla, Lydia T. Madsen, Joyce Dains

**Affiliations:** The University of Texas MD Anderson Cancer Center, Houston, Texas

## Abstract

Among patients with cancer, many factors can cause severe and persistent neutropenia, leading to increased morbidity and mortality. For patients with neutrophil deficiency, replacement with granulocyte transfusion (GTX) seems a rational approach. However, existing data on the efficacy of GTX have been inconclusive, and such adverse effects as respiratory distress and death indicate the need for further investigation into its efficacy. The purpose of this literature review was to address the question, "Are granulocyte transfusions effective in the management of adult oncology patients with neutropenia?" The focus was on adequate dosing, optimal timing of initiation, and adverse effects. Implications for practice for the provider and the niche population of neutropenic adult oncology patients that might benefit from GTX are presented.

In patients with cancer, several factors may cause severe and persistent neutropenia, including intense chemotherapy regimens, underlying malignancy, and stem cell transplantation as treatment. White blood cell (WBC) counts typically reach a nadir 7 to 10 days after chemotherapy administration, increasing the risk of a compromised immune system and susceptibility to opportunistic and life-threatening infections (Ang & Linn, 2011; Harris, 2009). Severe neutropenia increases the risk for fungal and bacterial infections, with the highest risk for patients with an absolute neutrophil count (ANC) < 500 cells/µL.

Granulocyte transfusion (GTX) is intended to restore granulocyte counts and theoretically decrease the risk of infection in neutropenic patients (Ang & Linn, 2011). Sepsis or infection in immunocompromised patients with cancer prompted experimentation with GTX in the early 1930s (Ang & Linn, 2011; Cherif, Axdorph, Kalin, & Bjorkholm, 2013; Stanworth et al., 2005 [assessed as up-to-date: June 29, 2010]). The first documented use of GTX in humans occurred in the 1960s, when granulocytes were collected from patients with chronic myeloid leukemia who had high WBC counts, using filtration methods that did not test for ABO compatibility or communicable diseases (Harris, 2009; Safdar, Rodriguez, Zuniga, Al Akhrass, & Pande, 2014; Stanworth et al., 2005 [assessed as up-to-date: June 29, 2010]).

Since the introduction of GTX, there have been many advances in collection and administration techniques. Contemporary practice is collection of granulocytes from related or unrelated donors, after priming the donor with a combination of granulocyte colony-stimulating factor (G-CSF) and corticosteroids for optimal granulocyte yield (Ang & Linn, 2011; Kim et al., 2011; Marfin & Price, 2013; Safdar et al., 2014; Seidel et al., 2008). However, no consensus on the benefits of GTX exists, although GTX is used as an adjunct therapy for the treatment of neutropenia (Kim et al., 2011; Raad et al., 2013; Safdar et al., 2014).

This literature review addresses the PICO (patient problem or population, intervention, comparison, outcome) question, "Are granulocyte transfusions effective in the management of adult oncology patients with neutropenia?" The role of the provider in the care of neutropenic patients receiving GTX and the niche population that might benefit from GTX are also discussed.

## METHODS

A literature search on granulocyte transfusions and neutropenic patients was performed, in conjunction with a medical librarian, using PubMed, Scopus, Ovid Medline, Cochrane Library, and the Cumulative Index to Nursing and Allied Health Literature (CINAHL) databases. Medical subject heading terms included "granulocytes," "leukocyte transfusion," "neutropenia," "aspergillus," "lung disease, fungal," "bacterial infections and mycoses," and "virus diseases." Items not published in English and those dealing with only animals were excluded, leaving 109 citations.

Review articles, case studies, editorials, abstracts, and conference presentations, as well as pediatric studies, studies in the non-oncology patient population, and studies in which the outcome results of GTX were not the primary or secondary outcome measure were also excluded. To delineate the usefulness of GTX in neutropenic patients in a contemporary setting, articles published between June 29, 2010, and December 31, 2014, were the time frame for this review. Referenced in-text citations were included if, on review, they contained relevant data and met inclusion/exclusion criteria. Six articles met all established inclusion and exclusion criteria.

## RESULTS

The results are organized by adverse effects and efficacy of GTX based upon its indication (bacterial vs. fungal infection), dose, and timing of administration. A summary of published studies on GTXs based on these results is presented in the Table beginning on page 413.

**Table:**
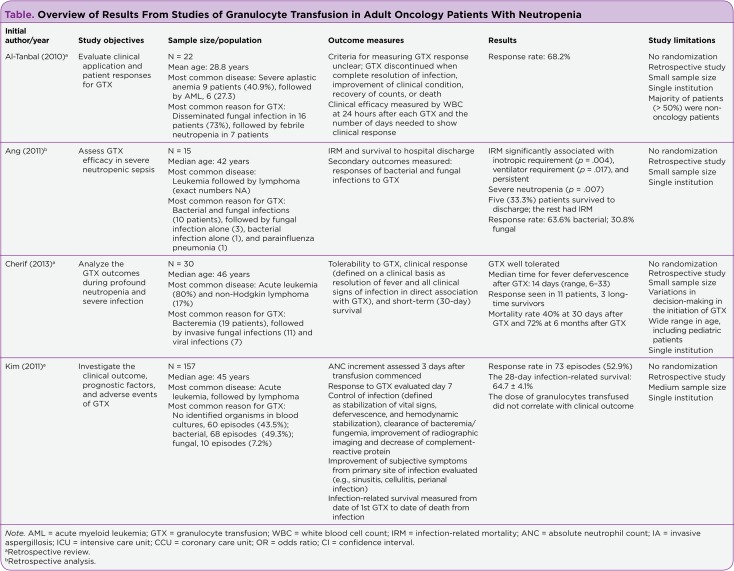
Overview of Results From Studies of Granulocyte Transfusion in Adult Oncology Patients With Neutropenia

**Table:**
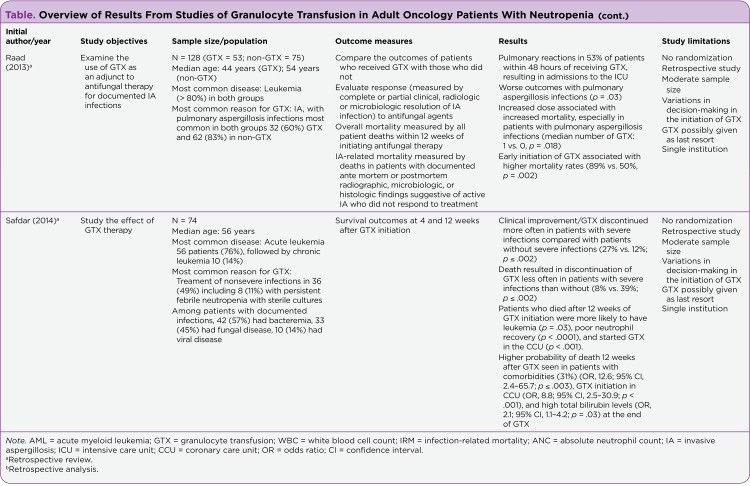
Overview of Results From Studies of Granulocyte Transfusion in Adult Oncology Patients With Neutropenia (cont.)

## ADVERSE EFFECTS

*Indication for GTX (fungal vs. bacterial infection)*: Raad et al. (2013) reported on a 128-patient cohort with fungal infections, noting that patients who received GTX had worse outcomes than the non-GTX group. Overall, worse outcomes, specifically transfusion-related acute lung injury (TRALI) and mortality were especially apparent in the patients with pulmonary invasive aspergillosis infections who had a poor response to antifungal therapy (*p* = .03). The authors of that study concluded that GTX should be used with caution or avoided completely in patients with known pulmonary fungal infection in the setting of hematologic malignancy and prolonged neutropenia (Raad et al., 2013).

In contrast, a retrospective review of 157 patients with febrile neutropenia conducted by Kim et al. (2011) showed that patients with fungal infections and Gram-negative bacterial infections had better infection control than patients with Gram-positive bacteremia and multiple species bacteremia (*p* = .019).

**TRALI and Other Respiratory Adverse Effects**

Ang and Linn (2011) reported no episodes of severe respiratory complications or other complications from GTX in their 15-patient cohort with either bacterial or fungal infections. Similarly, Cherif et al. (2013) reported that in a 30-patient cohort with bacterial, fungal, and viral infections, GTX was well tolerated with no adverse events (including respiratory manifestations) reported. In contrast, Raad et al. (2013) reported worsening shortness of breath and/or pulmonary infiltrates among 53% of patients within 48 hours of receiving GTX, resulting in admission to the intensive care unit (ICU).

Al-Tanbal et al. (2010) reported on 22 patients in whom fungal infection was the most common indication for GTX (16 patients, 73%). Overall, reported adverse effects were low, and the majority of patients (15 patients, 68.2%) had resolution of infection. One patient (4.5%) experienced TRALI and eventually died as a result of GTX. Notably, this patient had lung infiltrates with worsening lung function and multiorgan failure at the time of GTX initiation.

Safdar et al. (2014) retrospectively reviewed outcomes for 74 patients with fungal, bacterial, and viral infections in addition to patients with neutropenic fever of unknown origin. Adverse events were observed in 8 patients (11%), of whom 6 (8%) experienced respiratory complications.

Similar results were reported by Kim et al. (2011), where pneumonia was the most common infection, occurring in 54 episodes (39.1%), followed by localized infections in 35 episodes (25.5%). Adverse events of fever, hypotension, rash, and rigor were generally well tolerated; however, serious respiratory complications such as massive hemoptysis (3.5%) and respiratory failure requiring ventilator support (5.9 %) were reported. The presence of azotemia (serum creatinine > 1.5× upper limit of normal [ULN]) and prior pneumonia infiltration were associated with increased serious respiratory complications.

**Mortality**

In their 15-patient cohort, Ang and Linn (2011) reported overall survival rates of 33.3%, with the remaining patients having infection-related mortality. Mortality rates were higher among patients with bacterial infections (*p* = .077) and among those with increased age (median, 56 years; *p* = .141). No deaths were associated with factors related to GTX, type of disease, disease status, or treatment history, but rather infection-related mortality was significantly associated with inotropic requirement (*p* = .004), ventilator requirement (*p* = .017), and persistent severe neutropenia (*p* = .007).

Similar data by Cherif et al. (2013) indicated that mortality rates were high, with short-term (30 days post GTX) mortality of 40% and long-term (6 months post GTX) mortality of 72%, due to poor prognosis from the malignancy rather than GTX failure or poor infection control (p value not provided).

Kim et al. (2011) reported that septic shock (hazard ratio [HR], 4.62, 95% confidence interval [CI], 2.07–10.27; *p* < .001) and pneumonia/multiple primary infection sites (HR, 6.91/12.42, 95% CI, 2.21–21.67/3.34–46.17; *p* = .001/< .001) were significantly associated with failure to achieve infection control. The 28-day infection-related survival rate was 64.7 ± 4.1%. Refractory underlying disease (HR, 2.88, 95% CI, 1.83–4.52; *p* < .001) and septic shock (HR, 2.00, 95% CI, 1.32–3.04; *p* = .001) were significantly associated with poor survival rates.

In contrast, in their retrospective review of 74 patients, Safdar et al. (2014) found that mortality rates at 4 weeks were higher in patients without severe infections (57% vs. 26% with severe infections; *p* ≤ .001). Mortality rates at 12 weeks were higher in patients with leukemia (*p* = .03), in those without recovery of neutrophil counts (*p* < .0001), and in those who started GTX in the ICU (p < .001). Increased probability of death at 12 weeks was seen in patients with comorbidities (31%; OR, 12.6, 95% CI, 2.4–65.7; *p* ≤ .003), GTX started in the ICU (OR, 8.8, 95% CI, 2.5–30.9; *p* < .001), and high total bilirubin at the end of GTX (OR, 2.1, 95% CI, 1.1–4.2; *p* = .03).

Moreover, data by Raad et al. (2013) revealed a significantly higher mortality rate (*p* = .009) in the GTX group, especially those with pulmonary invasive aspergillosis infections (*p* = .03), compared with the non-GTX group. The Raad et al. (2013) findings were consistent with those of other studies, with higher mortality rates seen among the invasive aspergillosis patients when the patients had persistent neutropenia (p = .001), shortness of breath at baseline (*p* = .003), ICU stay (*p* < .0001), or treatment with GTX (*p* = .011).

**Dose of GTX**

Al-Tanbal et al. (2010) reported that one patient experienced TRALI after receiving a higher dose of GTX (> 1 × 1,010 granulocytes). Similarly, Raad et al. (2013) reported that patients who received an increased number of GTX (median number of GTX: 1 vs. 0; *p* = .018) had higher mortality and morbidity rates, as evidenced by worsening shortness of breath or worsening pulmonary infiltrates among 53% of patients. This finding was more apparent among patients with pulmonary invasive aspergillosis infections (Raad et al., 2013).

In the retrospective review conducted by Kim et al. (2011), respiratory complications of GTX, primarily seen in patients with pneumonic lung infiltration (*p* = .046) and septic shock (*p* = .188), did not correlate with the dose of GTX administered but rather with the volume in which the GTX was ad-ministered. High volumes of GTX (> 250 cc/day; *p* = .002) and azotemia (with increased creatinine of 1.5× ULN; *p* = .026) were associated with fluid overload and associated respiratory adverse effects, poor outcomes, and increased mortality.

**Timing of GTX Initiation**

Raad et al. (2013) observed higher mortality rates with early initiation of GTX, particularly among patients who received GTX within 1 week after antifungal therapy initiation (89% vs. 50%, *p* = .002). Additionally, Ang and Linn (2011), Kim et al. (2011), and Safdar et al. (2014) all reported increased mortality rates when GTX was delayed until patients were in the ICU, hemodynamically unstable, had multiorgan failure, or had refractory underlying disease. Ang and Linn (2011) and Safdar et al. (2014) also reported higher mortality rates for patients in whom GTX was initiated later, specifically in those requiring ventilator support and inotropes during GTX. In particular, data from Safdar et al.(2014) demonstrated that patients who received GTX in the ICU or while requiring ventilator support had a ninefold increased probability of death (*p* < .001).

## EFFICACY OF GTX

*Indication for GTX (fungal vs. bacterial infection)*: Ang and Linn (2011) reported better response rates to bacterial infections (63.6%) than to fungal infections (30.8%). Conversely, Kim et al. (2011) reported better fungal infection control observed in 73 (52.9%) of 138 episodes: 16 (47.1%) of 34 patients recovered from their infection. However, Al-Tanbal et al. (2010), Cherif et al. (2013), and Safdar et al. (2014) did not detect differences based on etiology of infection and provided evidence that GTX was more beneficial in patients with severe neutropenia and complicated infections than in patients without severe infections.

Al-Tanbal et al. (2010) reported the benefit of GTX in 15 (68%) of 22 severely neutropenic patients. Cherif et al. (2013) noted that 11 (37%) of 30 severely ill patients benefited from GTX, with 3 patients becoming long-time (> 5 years) survivors. Data presented by Safdar et al. (2014) showed that etiology of infection was not a predictor of fever resolution: Patients with either established fungal infections or established bacterial infections had a better response than patients with neutropenic fever of an unknown origin.

**Dose of GTX**

For GTX to be even marginally effective, a minimum dose of at least 1 × 1,010 granulocytes should be transfused (Al-Tanbal et al., 2010; Stanworth et al., 2005 [assessed as up-to-date: June 29, 2010]). Ang and Linn (2011) reported a positive correlation between WBC dose/kg and ANC increment (*p* = .013). Similarly, Kim et al. (2011) found that GTX dose significantly correlated with ANC increment (Pearson’s correlation coefficient, 0.178; *p* = .037). However, neither GTX dose nor ANC increment was associated with improved infection control or reduced mortality. Similarly, in a small retrospective review of 30 patients, Cherif et al. (2013) noted no significant difference in overall outcomes based on the dose of GTX (*p* > .05). In contrast, Raad et al. (2013) reported a correlation between higher doses of GTX and increased mortality and TRALI (*p* = .018).

**Timing of GTX Initiation**

Cherif et al. (2013) reported that patients who benefited from GTX had a shorter duration of neutropenia prior to treatment with GTX (mean 15 ± 6 days vs. 28 ± 16 days; *p* = .015). Comparably, Safdar et al. (2014) noted resolution of infection in 34 (46%) of 74 patients, with the remaining patients dying of advanced disease (22 patients, 30%) or infection-related causes (17 patients, 23%). Granulocyte transfusion was discontinued as a result of clinical improvement more often in patients with severe infections (27% vs. 12%) than without severe infections (*p* ≤ .002), whereas GTX was discontinued as a result of death more often in patients without severe infections (39% vs. 8%) than with severe infections (*p* = .002; Safdar et al., 2014).

## SUMMARY OF FINDINGS

Data presented indicate that GTX is most effective in adult oncology patients with neutropenia when initiated early in the disease course before clinical deterioration. This deterioration includes hemodynamic instability, multiorgan failure, impending ICU admission, or ventilator support. The prospect of neutrophil recovery is also a consideration. Granulocyte transfusion should be used with caution in patients with respiratory fungal infections.

Adverse effects or outcomes, based on the etiology of infection (fungal vs. bacterial), varied among study results. The majority of studies demonstrated that GTX is generally safe for use in neutropenic patients regardless of the etiology of infection (Al-Tanbal et al., 2010; Cherif et al., 2013; Kim et al., 2011; Safdar et al., 2014). Overall adverse effects of GTX were low; however, severe adverse effects associated with GTX were TRALI and death. Notably, each study reported that most adverse effects and poor outcomes were seen when patients had a poor recovery of neutrophil counts, poor prognosis for their malignancy, and multiple comorbidities (Al-Tanbal et al., 2010; Ang & Linn, 2011; Cherif et al., 2013; Kim et al., 2011; Marfin & Price, 2013; Safdar et al., 2014).

## DISCUSSION

Specific guidelines for the use of GTX in adult oncology patients with neutropenia do not currently exist because patients generally recover from neutropenia with the use of antibiotics and supportive care (Harris, 2009). Patients generally tolerate GTX well, with routine premedications not indicated. However, patients receiving GTX should be closely monitored for serious adverse effects, which often occur within a few hours of initiation (Marfin & Price, 2013). When patients experience allergic or febrile reactions such as fever, rash, chills, and hypotension, reactions can be managed with premedication using acetaminophen or corticosteroids in addition to slowing the infusion to prevent recurrences. Granulocyte transfusion should be discontinued when reactions are severe (Cherif et al., 2013; Harris, 2009; Raad et al., 2013).

Although rare, some serious adverse effects can be fatal. Pulmonary complications such as severe dyspnea, hypoxia, and pulmonary infiltrates are the most serious adverse effects of GTX and occur in about 5% to 10% of cases (Marfin & Price, 2013). Most of these pulmonary complications can be attributed to TRALI and appear to be most evident in patients with fungal infections (Raad et al., 2013).

Raad et al. (2013) suggest that patients with pulmonary infections are at higher risk for severe adverse effects and outcomes, including TRALI and death, than are patients with nonpulmonary infections. Patients with pulmonary aspergillosis infections had worse outcomes than did the non-GTX group. This risk may be related to the abnormal sequestration of granulocytes to the lungs after potential exposure to alloantibodies (Raad et al., 2013). This phenomenon was first described in the experiments conducted by Brecher, Wilbur, and Cronkite (1953) in the 1950s on neutropenic dogs, in which granulocytes migrated to the site of infection (Raad et al., 2013). Given this logic, when granulocytes migrate to the site of a pulmonary infection, they have the potential to cause TRALI, which may result in death. However, if granulocytes indeed migrate to the infection site, the number of circulating granulocytes decreases, potentially resulting in poor ANC increments despite adequate doses of GTX administration (Marfin & Price, 2013).

An increase in ANC levels can potentially shorten the duration of neutropenia and decrease the risks associated with neutropenia, particularly in patients with ANC < 500 cells/L. Our review found that most of the studies used the recommended dose of 1 × 1,010 granulocytes or higher. However, GTX dose and ANC increment did not appear to be associated with better clinical outcomes or improved mortality rates. Notably, Kim et al. (2011) found GTX to be associated with TRALI and higher mortality rates, with the association attributed to a combination of higher volumes of GTX (> 250 cc/day) and poor renal function. In such instances, administration of diuretics has the potential to proactively prevent respiratory distress related to fluid overload (Kim et al., 2011).

The majority of studies reviewed yielded inconclusive and conflicting data. These findings may be attributed to the following limitations: the retrospective nature of the studies; no randomization of patient cohorts; and multiple variations in the timeline and dosing decisions when initiating GTX by the provider, resulting in many patients receiving GTX as a last resort. Across the studies, many patients were critically ill, in the ICU, and in need of ventilator support when GTX was initiated, which may have caused a bias disfavoring GTX. Additionally, sample sizes were small, with many studies conducted outside of the United States in locations where practice guidelines for the management of neutropenic patients may have differed.

## IMPLICATIONS FOR ADVANCED PRACTITIONERS

Implications for advanced practitioners include careful screening of neutropenic patients prior to the initiation of GTX, especially in critically ill neutropenic patients with pulmonary infections. Risks associated with GTX are generally low but can result in severe respiratory complications and even death. Conversely, GTX can decrease the duration of neutropenia as well as prevent infections and other complications related to prolonged neutropenia in adult oncology patients. Additionally, GTX is expensive, difficult to obtain, not always feasible, associated with inconclusive data, and above all, can be detrimental if given to the wrong patient.

Despite such barriers, GTX may be beneficial in neutropenic adult oncology patients if initiated early, while patients are hemodynamically stable and before multiorgan failure, ICU admission, or ventilator support. Granulocyte transfusion should be used with caution in patients with respiratory infections.

## CONCLUSION

Despite conflicting data, GTX appears beneficial in the management of neutropenic adult oncology patients. The key is a tailored rather than generalized approach when it comes to the selection of patients. There is a need to establish proper guidelines with inclusion and exclusion criteria and a need for further research in this area.
